# Neurological and neuropsychiatric disorders associated with COVID-19. Part II: neuropsychiatric disorders and final consideration

**DOI:** 10.31744/einstein_journal/2021CE6464

**Published:** 2021-10-20

**Authors:** Martina Giacalone, Marcos Roberto Tovani-Palone, Luca Marin, Massimiliano Febbi, Tommaso Russano, Andrea Giacalone

**Affiliations:** 1 Faculty of Medicine and Pharmacy, Medicine and Surgery Sapienza University of Rome Rome Italy Faculty of Medicine and Pharmacy, Medicine and Surgery, Sapienza University of Rome, Rome, Italy.; 2 Faculdade de Medicina de Ribeirão Preto Universidade de São Paulo Ribeirão PretoSP Brazil Faculdade de Medicina de Ribeirão Preto, Universidade de São Paulo, Ribeirão Preto, SP, Brazil.; 3 Department of Rehabilitation Faculty of Medicine University of Ostrava Ostrava Czech Republic Department of Rehabilitation, Faculty of Medicine, University of Ostrava, Ostrava, Czech Republic.; 4 Faculty of Medicine and Psychology Sant’Andrea Hospital Sapienza University of Rome Rome Italy Department of Neuroscience, Mental Health and Sensory Organs (NESMOS), Faculty of Medicine and Psychology, Sant’Andrea Hospital, Sapienza University of Rome, Rome, Italy.; 5 Department of Industrial Engineering, Technologies for Sports Medicine and Rehabilitation University of Rome Tor Vergata Rome Italy Department of Industrial Engineering, Technologies for Sports Medicine and Rehabilitation, University of Rome Tor Vergata, Rome, Italy.

Dear Editor,

Coronavirus disease 2019 (COVID-19) patients may experience mental confusion, agitation, symptoms of depression, anxiety, and insomnia.^(1)^ Several risk factors have been considered regarding neuropsychiatric complications of COVID-19, many of which are already known to be associated with other mental health problems.^(2)^

The psychological status of COVID-19 patients may be worsened by different aspects, such as quarantine, isolation, fear about the transmission of the virus, and concern about the possibility of complication of the disease.^(3)^ Moreover, higher levels of stress have been reported in hospitalized COVID-19 patients compared with general population.^(4)^ For this reason, constant monitoring of these patients is essential, both psychologically and clinically. Neuropsychological sequelae may lead to avoidance and detachment from others, preclinical symptoms of post-traumatic stress disorder, and suicidal behavior, which represent conditions that may result in long-term psychiatric illnesses.^(5)^ In this sense, whenever possible, psychiatric care should be provided through telemedicine. However, there may be cases where a face-to-face consultation with the patient is necessary, such as in situations of psychiatric emergency, risk of psychiatric relapse, or new emergent cases of severe mental illness.^(6)^ Additionally, social media platforms (*e.g.* WeChat and Weibo) can be used to share strategies, guidelines, and educational programs for managing potential mental distress.^(7)^

Another important aspect in this discussion is the fact that mental illnesses have a significant impact on general health as well as in social and individual wellbeing. Thus, it becomes essential to integrate psychological support through specific protocols to standard medical care in order to ensure the best quality of care for these patients and their family.^(8)^

## Conclusions and prospects

Given the pandemic spread of COVID-19, the study of a wide range of clinical manifestations of the disease is of major importance. Despite the nervous system manifestations are being increasingly recognized in COVID-19 patients, there is limited studies published in the literature on this subject and often the lack of a systematic data collection makes any interpretation difficult. Hence, there is a need to adopt a systematic and methodical approach to better clarify the importance of viral neuroinvasion and its neurological and neuropsychiatric sequelae.

Another important issue raised by some studies is the understanding of the mechanisms underlying neuronal damage. In fact, it is uncertain whether the cell damage seen in patients who develop neurological manifestations, such as neuropathy, cerebrovascular disease, or disseminated acute encephalomyelitis, is caused by the host’s immune response to viral infection or by the virus itself.

In summary, to date, the most common neurological complications related to COVID-19 are the state of hypercoagulability and cerebrovascular discomfort; however, other less frequent manifestations, such as Guillain-Barré syndrome and myelitis have also been described. Although the number of COVID-19 patients presenting neurological manifestations is significant, it is considerably lower compared with those of patients with isolated respiratory symptoms. On the other hand, as the involvement of the central and peripheral nervous system can cause severe and irreversible disabilities, such cases may require long-term medical care, as well as economic and social support.^(9)^ Consequently, further careful clinical, diagnostic, and epidemiological studies are warranted to better define the neurological manifestations of COVID-19, in order to clarify the pathogenesis, guide the management, and estimate the burden of related neuropsychiatric sequelae, particularly for the many uncertainties that still exist about the disease.^(10)^
