# Supplementary material for: Neurological and neuropsychiatric disorders associated with COVID-19. Part II: neuropsychiatric disorders and final consideration
Source: Einstein (Sao Paulo). 2021 Oct 20;19:eCE6464. doi: 10.31744/einstein_journal/2021CE6464 (PMC8528448; doi:10.31744/einstein_journal/2021CE6464)
Supplement: Translation

# Translation: Distúrbios neurológicos e neuropsiquiátricos associados à COVID-19. Parte II: distúrbios neuropsiquiátricos e considerações finais

**Authors:** Martina Giacalone, Marcos Roberto Tovani-Palone, Luca Marin, Massimiliano Febbi, Tommaso Russano, Andrea Giacalone

**Affiliations:** 1 Italy Faculty of Medicine and Pharmacy, Medicine and Surgery, Sapienza University of Rome, Rome, Italy.; 2 Brasil Faculdade de Medicina de Ribeirão Preto, Universidade de São Paulo, Ribeirão Preto, SP, Brasil.; 3 Czech Republic Department of Rehabilitation, Faculty of Medicine, University of Ostrava, Ostrava, Czech Republic.; 4 Italy Department of Neuroscience, Mental Health and Sensory Organs (NESMOS), Faculty of Medicine and Psychology, Sant’Andrea Hospital, Sapienza University of Rome, Rome, Italy.; 5 Italy Department of Industrial Engineering, Technologies for Sports Medicine and Rehabilitation, University of Rome Tor Vergata, Rome, Italy.

Caro Editor,

Pacientes com a doença pelo coronavírus 2019 (COVID-19) podem apresentar confusão mental, agitação, sintomas de depressão, ansiedade e insônia.^(1)^ Diversos fatores de risco têm sido descritos para as complicações neuropsiquiátricas da COVID-19, muitos dos quais já são conhecidos por estarem associados com outros problemas de saúde mental.^(2)^

O estado psicológico de pacientes com COVID-19 pode ser agravado por diferentes aspectos, como a quarentena, o isolamento social, o medo sobre a transmissão do vírus e a preocupação sobre a possibilidade de complicações da doença.^(3)^ Além disso, altos níveis de estresse têm sido reportados em pacientes hospitalizados com COVID-19 comparados aos da população geral.^(4)^ Dessa forma, o monitoramento constante desses pacientes é essencial, tanto do ponto de vista psicológico quanto clínico. As sequelas neuropsicológicas podem levar ao comportamento de evitação e distanciamento, sintomas pré-clínicos de transtorno de estresse pós-traumático e a comportamento suicida. Essas condições podem por sua vez resultar em doenças psiquiátricas de longa duração.^(5)^ Nesse sentido, sempre que possível, a assistência psiquiátrica deve ser disponibilizada por meio da telemedicina. Todavia, há casos em que a consulta presencial com o paciente é indispensável, como em situações de emergência psiquiátrica, risco de recaída psiquiátrica ou de novos casos emergentes de doença mental grave.^(6)^ Também, plataformas de mídia social (por exemplo: WeChat e Weibo) podem ser utilizadas para compartilhar estratégias, diretrizes e programas educacionais para manejo de distúrbios mentais relacionados.^(7)^

Outro ponto importante é o fato de que as doenças mentais têm impacto significativo na saúde geral, bem como no bem-estar social e individual dos pacientes. Logo, é imprescindível integrar o suporte psicológico ao atendimento médico padrão por meio de protocolos específicos, a fim de garantir melhor qualidade de atendimento para os pacientes e seus familiares.^(8)^

## Conclusões e perspectivas

Dada a propagação da pandemia da COVID-19, é de valiosa importância o estudo da ampla variedade de manifestações clínicas da doença. Apesar das manifestações do sistema nervoso serem cada vez mais reconhecidas em pacientes com COVID-19, ainda há poucos estudos na literatura sobre o assunto. Além disso, a falta de uma coleta sistemática de dados torna muitas vezes qualquer interpretação difícil. Desse modo, há necessidade de se adotar uma abordagem sistemática e metódica para melhor explicar a magnitude da neuroinvasão viral e as sequelas neurológicas e neuropsiquiátricas relacionadas.

Outra questão importante levantada por alguns estudos é a necessidade de compreender os mecanismos subjacentes aos danos neuronais. De fato, não se conhece se o dano celular observado em pacientes que desenvolvem manifestações neurológicas, como neuropatia, doença cerebrovascular ou encefalomielite disseminada aguda, seja causado pela resposta imune do hospedeiro à infeção viral ou pelo próprio vírus.

Em resumo, até o momento, as complicações neurológicas mais comuns associadas à COVID-19 são o estado de hipercoagulabilidade e o desconforto cerebrovascular. Contudo, outras manifestações menos frequentes, como a síndrome de Guillain-Barré e mielite, também têm sido descritas. Embora o número de pacientes com COVID-19 apresentando manifestações neurológicas seja significativo, tal número é consideravelmente menor em comparação ao de pacientes com sintomas respiratórios isolados. Por outro lado, como o envolvimento do sistema nervoso central e periférico pode causar incapacidades graves e irreversíveis, tais casos devem, por vezes, requerer tratamento médico de longo prazo, bem como suporte econômico e social.^(9)^ Assim, são necessários mais estudos clínicos, diagnósticos e epidemiológicos criteriosos para melhor definir as manifestações neurológicas da COVID-19, buscando elucidar sua patogênese, orientar o manejo de pacientes e estimar a carga de sequelas neuropsiquiátricas associadas, especialmente em vista das muitas incertezas que ainda existem sobre a doença.^(10)^
